# Prehospital and emergency department airway management of severe penetrating trauma in Sweden during the past decade

**DOI:** 10.1186/s13049-023-01151-4

**Published:** 2023-11-24

**Authors:** Mattias Renberg, Martin Dahlberg, Mikael Gellerfors, Elham Rostami, Mattias Günther

**Affiliations:** 1 Department of Anesthesiology and Intensive Care, Södersjukhuset, Sjukhusbacken, 10, S1 SE-118 83 Stockholm, Sweden; 2 Department of Surgery, Södersjukhuset, Karolinska Institutet, Stockholm, Sweden; 3 Department of Perioperative Medicine and Intensive Care, Karolinska University Hospital, Stockholm, Sweden; 4 Rapid Response Car, Capio, Stockholm, Sweden; 5 Department of Physiology and Pharmacology, Karolinska Institutet, Stockholm, Sweden; 6 Swedish Air Ambulance (SLA), Mora, Sweden; 7 Experimental Traumatology Unit, Department of Neuroscience, Karolinska Institutet, Stockholm, Sweden; 8 Department of Medical Sciences, Neurosurgery, Uppsala University, Uppsala, Sweden; 9 Department of Clinical Science and Education, Södersjukhuset, Karolinska Institutet, Stockholm, Sweden

**Keywords:** Prehospital, Airway management, Intubation, Penetrating trauma, Trauma

## Abstract

**Background:**

Prehospital tracheal intubation (TI) is associated with increased mortality in patients with penetrating trauma, and the utility of prehospital advanced airway management is debated. The increased incidence of deadly violence in Sweden warrants a comprehensive evaluation of current airway management for patients with penetrating trauma in the Swedish prehospital environment and on arrival in the emergency department (ED).

**Methods:**

This was an observational, multicenter study of all patients with penetrating trauma and injury severity scores (ISSs) ≥ 15 included in the Swedish national trauma register (SweTrau) between 2011 and 2019. We investigated the frequency and characteristics of prehospital and ED TI, including 30-day mortality and patient characteristics associated with TI.

**Result:**

Of 816 included patients, 118 (14.5%) were intubated prehospitally, and 248 (30.4%) were intubated in the ED. Patients who were intubated prehospitally had a higher ISS, 33 (interquartile range [IQR] 25, 75), than those intubated in the ED, 25 (IQR 18, 34). Prehospital TI was associated with a higher associated mortality, OR 4.26 (CI 2.57, 7.27, *p* < 0.001) than TI in the ED, even when adjusted for ISS (OR 2.88 [CI 1.64, 5.14, *p* < 0.001]). Hemodynamic collapse (≤ 40 mmHg) and low GCS score (≤ 8) were the characteristics most associated with prehospital TI. Traumatic cardiac arrests (TCAs) occurred in 154 (18.9%) patients, of whom 77 (50%) were intubated prehospitally and 56 (36.4%) were intubated in the ED. A subgroup analysis excluding TCA showed that patients with prehospital TI did not have a higher mortality rate than those with ED TI, OR 2.07 (CI 0.93, 4.51, *p* = 0.068), with OR 1.39 (0.56, 3.26, *p* = 0.5) when adjusted for ISS.

**Conclusion:**

Prehospital TI was associated with a higher mortality rate than those with ED TI, which was specifically related to TCA; intubation did not affect mortality in patients without cardiac arrest. Mortality was high when airway management was needed, regardless of cardiac arrest, thereby emphasizing the challenges posed when anesthesia is needed. Several interventions, including whole blood transfusions, the implementation of second-tier EMS units and measures to shorten scene times, have been initiated in Sweden to counteract these challenges.

**Supplementary Information:**

The online version contains supplementary material available at 10.1186/s13049-023-01151-4.

## Introduction

Trauma is a leading cause of mortality and morbidity in the young and healthy.[1, 2] Gunshot wounds (GSWs) and stab wounds (SWs) constitute up to 20 percent of all trauma in the US.[3] The incidence of penetrating trauma is lower in western Europe, accounting for approximately 10 percent of all injuries.[3, 4] The incidence of homicide and gun homicide has increased in Sweden in the past decade and is now well above the European average, despite a decline in the majority of European countries.[5] .

Delay, or failure in securing an airway is a preventable cause of death in trauma patients,[6–8] and emergency airway management is more challenging when needed outside the operating theater. Consequently, prehospital and out-of-theater emergency tracheal intubations (TI) may be associated with a lower success rate and a higher incidence of complications than TI in operating theatres.[9, 10] Nevertheless, prehospital TI can meet the standard of in-hospital emergency intubations when performed by experienced airway providers.[9, 11] In addition to risks with TI, anesthesia drugs and positive pressure ventilation may increase mortality in hypovolemic patients. [12–14] Positive pressure ventilation leads to an increased intra-thoracic pressure, which may decrease venous return and subsequently cardiac output in a hypovolemic state. Trauma patients are heterogeneous, and optimal airway management depends on the patient’s specific condition, airway provider experience and environmental factors.[3, 8, 15] TI in patients with penetrating trauma is associated with increased mortality,[9, 16, 17] specifically in patients with hemorrhagic shock.[12, 13] The increased incidence of deadly violence in Sweden warrants a comprehensive evaluation of current airway management for patients with penetrating trauma in the prehospital environment and in the ED.

The aims of this study were to present the characteristics associated with TI, its associated causes of mortality and to compare prehospital and ED intubations in patients with penetrating trauma in Sweden between 2011 and 2019, during which time the incidence of gun violence increased.[18] We analyzed data from the nationwide trauma registry in Sweden (SweTrau) and hypothesized that prehospital TI was associated with increased mortality.

## Methods

### Study population

**Fig. 1:**
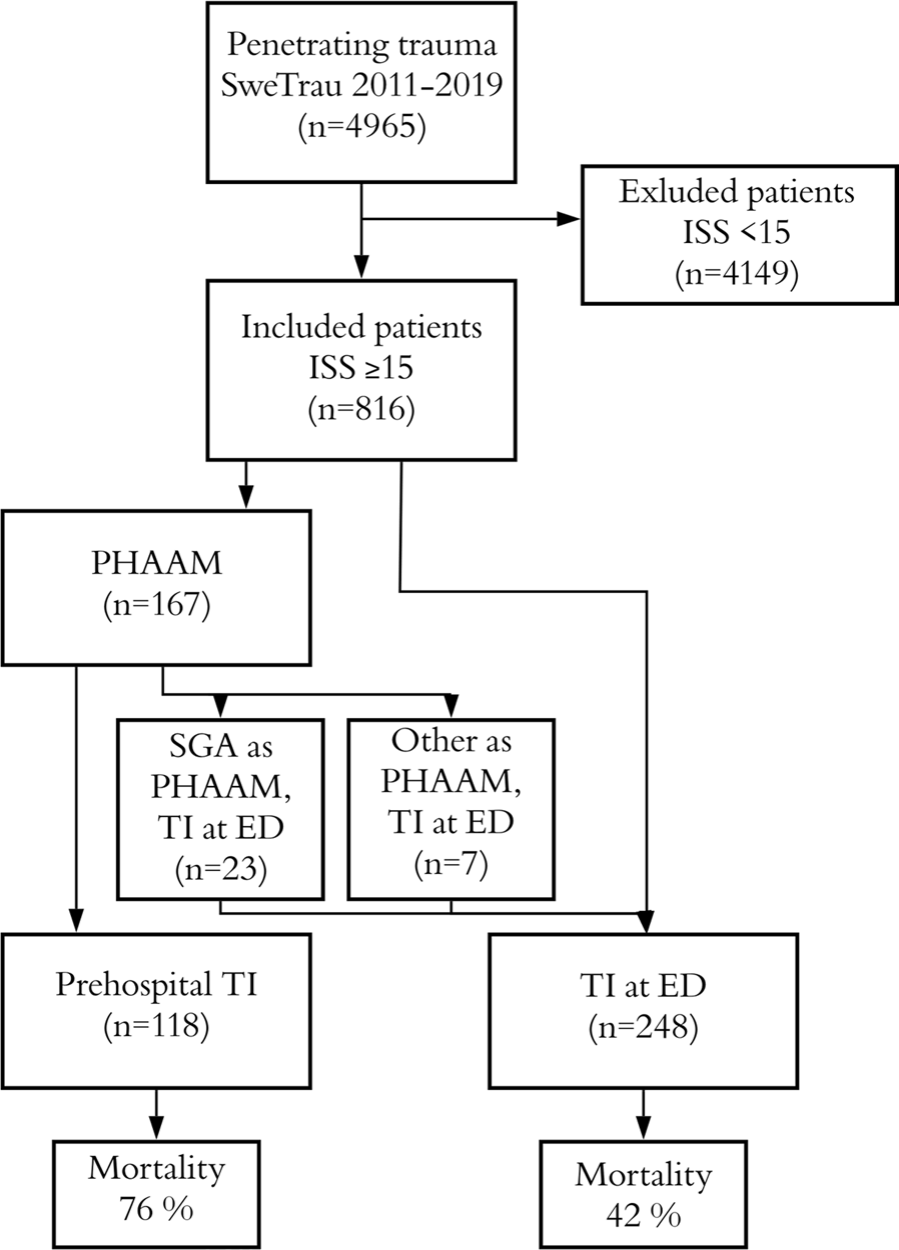
Flowchart of patient inclusion. Abbreviations: ED = emergency department, ISS = injury severity score, PHAAM = prehospital advanced airway management, SGA = supraglottic airway device, SweTrau = Swedish national trauma registry, TI = tracheal intubation

This was a retrospective, descriptive multicenter study of all patients with penetrating trauma and an injury severity score (ISS) ≥ 15 who were registered in SweTrau between its inception on June 13, 2011, and December 31, 2019 (Fig. 1). Patients of all ages and sexes were included. The population in Sweden was 9,415,570 persons in 2011 and 10,327,589 persons in 2019. The study was approved by the Swedish Ethical Review Authority (no 2019–02842) and by the SweTrau steering group. Patient consent was waived.

### Swedish trauma registry and participating hospitals

Data were extracted from the national trauma registry in Sweden called SweTrau. The SweTrau registry was established in 2011 and is based on “The Utstein Trauma Template for Uniform Reporting of Data Following Major Trauma”.[19] Hospitals with trauma capabilities (anesthesia, surgery, and radiology at all times) are eligible to contribute to SweTrau. In 2019, 46 out of 50 (92%) eligible hospitals were affiliated with SweTrau, and 43 (86%) of the participating hospitals actively registered data.[20] SweTrau estimates the registry’s coverage of trauma patients by comparing the number of patients requiring intensive care admission to the number of patients registered in the Swedish Intensive Care Registry (SIR) who were admitted with trauma and injury diagnoses SA01-TA04 and TA09-TA13. In SweTrau’s 2019 annual report, the coverage was estimated at 72.6%.[20] To be included in SweTrau, patients needed to have at least one of the following criteria: (i) exposure to a traumatic event that caused the receiving hospital to dispatch either a full or partial trauma team, (ii) an ISS > 15, even without the intervention of a trauma team, and (iii) an ISS > 15 when transferred to a participating hospital within 7 days of the traumatic event. The exclusion criteria were as follows: (i) the trauma team was dispatched without a precipitating traumatic event and (ii) the only traumatic injury was a chronic subdural hematoma.

### Definitions and data management

Penetrating trauma was defined as injuries sustained by sharp objects. TI and surgical airways were registered as TI in SweTrau. Bag-valve-mask ventilation and nasal or oropharyngeal airway adjuncts were not considered advanced airway management. Patients registered in SweTrau as intubated both prehospitally and in the ED were included as prehospital TI in the study. Patients who received a prehospital supraglottic airway device and were subsequently intubated in the ED were defined as TI in the ED. Patients registered with “other” as prehospital airway management and subsequently intubated in the ED were included as TI in the ED. There was no standard operating procedure for prehospital TI proposed by the county councils in Sweden during the study period that could apply to the entire dataset. General guidelines and data on prehospital anesthesia and advanced airway management of undifferentiated patients in a Scandinavian setting during the study period have been reported.[11, 21] The prehospital provider level is registered in SweTrau as (i) none—no medical training, (ii) basic—emergency medical technician, (iii) advanced without physician – nurse, (iv) advanced with physician—physician. In SweTrau, palpable pulses are registered as weak/strong radial, femoral, only carotid, or no carotid when systolic blood pressure (SBP) is missing. Transcription was performed to quantify palpated pulses for a multivariate analysis of characteristics associated with prehospital TI. The following conservative transcribed values were based on Deakin et al.: no carotid, ≤ 40 mmHg; only carotid, 50 mmHg; femoral, 60 mmHg; weak radial, 70 mmHg; clear radial, ≥ 80 mmHg).[22] A post hoc subgroup analysis was performed in which patients with traumatic cardiac arrest (TCA) were excluded from the dataset.

### Statistical analysis

Statistical analysis and data preparation were done with R (v 4.0.3). Data are presented as medians with interquartile ranges (IQRs) for continuous variables and numbers and percentages for categorical variables. Continuous data were compared using Wilcoxon rank sum test. Mortality associated with TI and ISS was tested using restricted cubic spline. Dichotomous variables were created for prehospital TI, Glasgow Coma Scale (GCS) score (3–8/9–15), hypo- and hyperventilation (respiratory rate [RR] ≤ 9/ > 29), and severe head and/or thoracic trauma (severity ≥ 3 of AIS code head and/or thorax) and used in a multivariable logistic regression analysis. The results were reported as ORs and 95% confidence intervals (CIs). Variables were selected based on patient characteristics with plausible associations with increased mortality. Statistical significance was set as *p* < 0.05. No power calculation was made due to the descriptive nature of the study.

## Results

The study analyzed 4965 patients with penetrating trauma between 2011 and 2019, and 816 patients (16.4%) met the inclusion criteria.

### Baseline characteristics

The median age was 29 years for patients with prehospital TI and 30 years for patients intubated in the ED (Table 1). Stab wounds were the most common injury mechanism for patients with prehospital TI (50%), followed by GSWs (44%). In patients with TI in the ED, SW was most prevalent (50%), followed by GSW (44%). The median ISS was 33 for patients with prehospital TI and 21 for those without TI, and the ISS was 25 for TI in the ED and 22 for those without TI. Prehospital TI was performed in 43.4% of patients with a GCS score ≤ 8, 21.2% with a GCS score 9–12 and 1.8% with a GCS score 13–15 (Table 2). A majority, 64% (*n* = 75), of prehospital TI were performed without medications, and 36% (*n* = 43) were performed with medications, compared with TI in the ED, where 84% (*n* = 208) of intubations were with medications and 16% (*n* = 40) were without medications (Table 3). The median scene time were significantly (*p* < 0.001) longer when prehospital TI were required (21 min [IQR 16, 36]) compared with patients without prehospital TI (12 min [IQR 7, 18]).

**Table 1:** Baseline characteristics

Characteristic	Prehospital airway management		ED airway management	
	TI, *n* = 118^*1*^	no TI, * n* = 567^*1*^	TI, * n* = 248^*1*^	no TI, * n* = 482^*1*^
Age (years)	29 (21, 41)	31 (23, 46)	30 (23, 43.5)	29 (22, 44)
Missing	0	1	1	0
Sex				
Female	14/118 (12%)	55/567 (9.7%)	18/248 (7.3%)	44/482 (9.1%)
Male	104/118 (88%)	512/567 (90%)	230/248 (93%)	438/482 (91%)
Injury mechanism				
GSW	52/118 (44%)	171/567 (30%)	109/248 (44%)	157/482 (33%)
SW	59/118 (50%)	351/567 (62%)	125/248 (50%)	283/482 (59%)
Other	7/118 (5.9%)	45/567 (7.9%)	14/248 (5.6%)	42/482 (8.7%)
ISS	33 (25, 75)	21 (17, 26)	25 (18, 34)	22 (17, 28.5)
Prehospital GCS score				
3–8	79/92 (86%)	103/463 (22%)	79/174 (45%)	90/338 (27%)
9–12	7/92 (7.6%)	26/463 (5.6%)	18/174 (10%)	11/338 (3.3%)
13–15	6/92 (6.5%)	334/463 (72%)	77/174 (44%)	237/338 (70%)
Missing	26	104	74	144
First blood pressure (mmHg)	0.0 (0.0, 80)	118 (97.75, 137)	110 (90, 130)	110 (85.5, 135)
Missing	50	227	159	204
First blood pressure (RTS)				
No carotid	16/39 (41%)	48/170 (28%)	34/88 (39%)	24/99 (24%)
Only carotid	8/39 (21%)	12/170 (7.1%)	10/88 (11%)	7/99 (7.1%)
Femoral	3/39 (7.7%)	12/170 (7.1%)	6/88 (6.8%)	7/99 (7.1%)
Weak radial	6/39 (15%)	42/170 (25%)	19/88 (22%)	26/99 (26%)
Clear radial	6/39 (15%)	56/170 (33%)	19/88 (22%)	35/99 (35%)
First respiratory rate (RTS)				
0	58/87 (67%)	60/497 (12%)	44/177 (25%)	68/353 (19%)
1–9	5/87 (5.7%)	5/497 (1.0%)	3/177 (1.7%)	6/353 (1.7%)
10–29	18/87 (21%)	327/497 (66%)	92/177 (52%)	215/353 (61%)
> 29	6/87 (6.9%)	105/497 (21%)	38/177 (21%)	64/353 (18%)
Missing	31	70	71	129
Prehospital airway provider				
None	0/118 (0%)	3/567 (0.5%)	27/235 (11%)	14/421 (3.3%)
Basic	1/118 (0.8%)	33/567 (5.8%)	5/235 (2.1%)	23/421 (5.5%)
Nurse	53/118 (45%)	468/567 (83%)	174/235 (74%)	305/421 (72%)
Physician	64/118 (54%)	63/567 (11%)	29/235 (12%)	79/421 (19%)
Missing			13	61
Prehospital transport				
Ground EMS	85/118 (72%)	518/567 (91%)	191/248 (77%)	357/482 (74%)
Helicopter EMS	33/118 (28%)	46/567 (8.1%)	17/248 (6.9%)	50/482 (10%)
Missing	0/118 (0%)	3/567 (0.5%)	19/248 (7.7%)	50/482 (10%)
Other			21/248 (8.5%)	25/482 (5.2%)

^*1*^*Median (IQR); n/N (%).* Abbreviations: ED = emergency department, EMS = emergency medical service, GCS = Glasgow coma scale, GSWs = gunshot wounds, IQR = interquartile range, SW = stab wounds

**Table 2:** Prehospital TI stratified by GCS score

Characteristic	3–8, *n* = 182			9–12, * n* = 33			13–15, * n* = 340		
	n	TI, * n* = 79^*1*^	no TI, * n* = 103^*1*^	n	TI, * n* = 7^*1*^	no TI, * n* = 26^*1*^	n	TI, * n* = 6^*1*^	no TI, *n* = 334^*1*^
First blood pressure (mmHg)	81	0 (0, 0)	112 (0, 136)	19	110 (100, 150)	96 (81, 114)	247	100 (100, 130)	120 (100, 139)
Unknown		30	71		2	12		3	90
First blood pressure (RTS)	182			33			340		
no carotid		11 (41%)	45 (79%)		0 (0%)	0 (0%)		0 (0%)	0 (0%)
only carotid		5 (19%)	3 (5.3%)		2 (100%)	2 (25%)		0 (0%)	3 (4.2%)
femoral		2 (7.4%)	2 (3.5%)		0 (0%)	2 (25%)		1 (33%)	2 (2.8%)
weak radial		5 (19%)	5 (8.8%)		0 (0%)	2 (25%)		0 (0%)	27 (38%)
clear radial		4 (15%)	2 (3.5%)		0 (0%)	2 (25%)		2 (67%)	40 (56%)
Respiratory rate (RTS)	149			29			316		
0		48 (75%)	55 (65%)		0 (0%)	0 (0%)		0 (0%)	0 (0%)
1–9		4 (6.2%)	3 (3.5%)		1 (20%)	1 (4.2%)		0 (0%)	0 (0%)
10–29		9 (14%)	23 (27%)		3 (60%)	12 (50%)		4 (80%)	244 (78%)
> 29		3 (4.7%)	4 (4.7%)		1 (20%)	11 (46%)		1 (20%)	67 (22%)
Unknown		15	18		2	2		1	23
30-day mortality	182			33			340		
Dead		65 (82%)	82 (80%)		3 (43%)	5 (19%)		1 (17%)	13 (3.9%)
Alive		13 (16%)	20 (19%)		4 (57%)	21 (81%)		5 (83%)	316 (95%)
Unknown		1 (1.3%)	1 (1.0%)		0 (0%)	0 (0%)		0 (0%)	5 (1.5%)

^*1*^*Median (IQR); n/N (%).* Abbreviations: GCS = Glasgow coma scale, IQR = interquartile range, RTS = revised trauma score, TI = tracheal intubation

**Table 3:** Outcomes

Characteristic	Prehospital airway management		ED airway management	
	TI, * n* = 118^*1*^	no TI, * n* = 567^*1*^	TI, * n* = 248^*1*^	no TI, * n* = 482^*1*^
Airway method				
Tracheal, meds	43/118 (36%)	0/49 (0%)	208/248 (84%)	0/27 (0%)
Tracheal, no meds	75/118 (64%)	0/49 (0%)	40/248 (16%)	0/27 (0%)
Supraglottic, meds	0/118 (0%)	8/49 (16%)	0/248 (0%)	3/27 (11%)
Supraglottic, no meds	0/118 (0%)	21/49 (43%)		
Other	0/118 (0%)	12/49 (24%)	0/248 (0%)	6/27 (22%)
Unknown	0/118 (0%)	8/49 (16%)	0/248 (0%)	18/27 (67%)
No airway management/missing	0	518	0	455
Ventilator days	3 (1, 5)	1 (1, 3)	1 (1, 4)	1 (1, 4)
No ventilator days/missing	69	299	67	319
30-day mortality				
Dead	90/118 (76%)	117/567 (21%)	105/248 (42%)	107/482 (22%)
Alive	26/118 (22%)	442/567 (78%)	139/248 (56%)	367/482 (76%)
Unknown	2/118 (1.7%)	8/567 (1.4%)	4/248 (1.6%)	8/482 (1.7%)
Glasgow outcome scale score				
1	90/116 (78%)	116/567 (20%)	106/248 (43%)	106/481 (22%)
2	0/116 (0%)	5/567 (0.9%)	4/248 (1.6%)	6/481 (1.2%)
3	13/116 (11%)	87/567 (15%)	45/248 (18%)	74/481 (15%)
4	10/116 (8.6%)	218/567 (38%)	62/248 (25%)	177/481 (37%)
5	2/116 (1.7%)	136/567 (24%)	28/248 (11%)	115/481 (24%)
Unknown	1/116 (0.9%)	5/567 (0.9%)	3/248 (1.2%)	3/481 (0.6%)
Missing	2	0	0	1

^*1*^*Median (IQR); n/N (%).* Abbreviations: ED = emergency department, IQR = interquartile range, SGA = supraglottic airway device, TI = tracheal intubation

### Outcomes

**Fig. 2:**
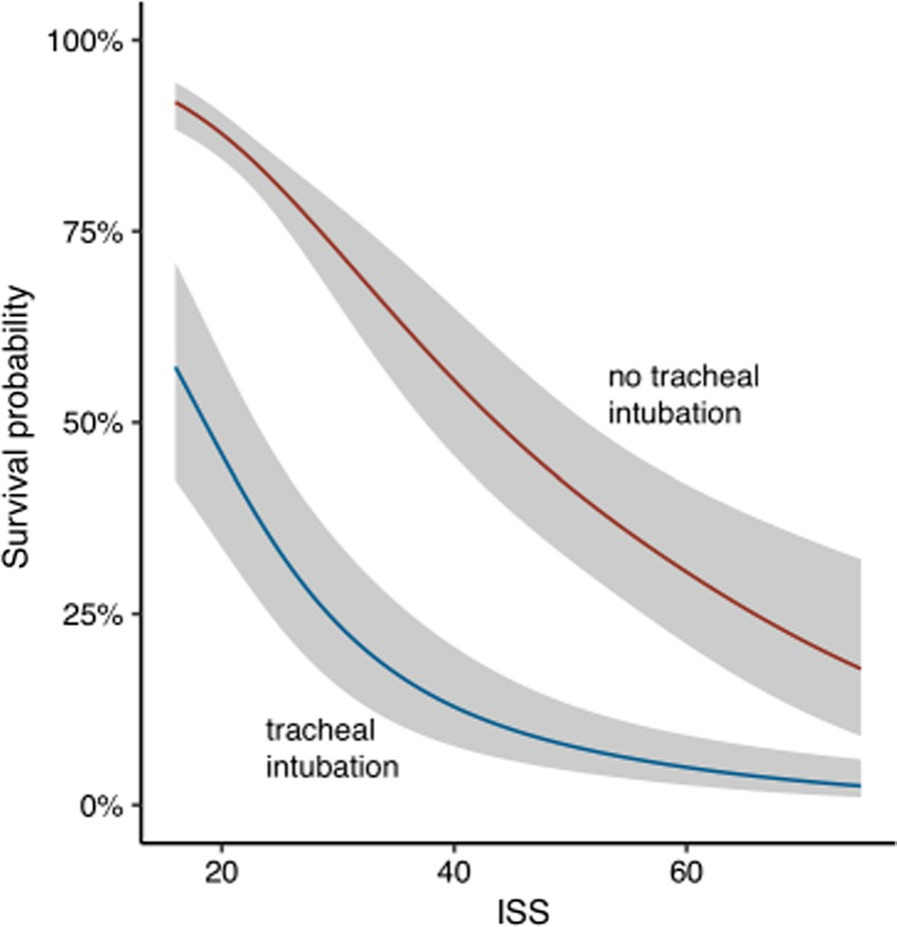
Mortality associated with prehospital TI and ISS. Abbreviation: ISS = injury severity score

**Fig. 3:**
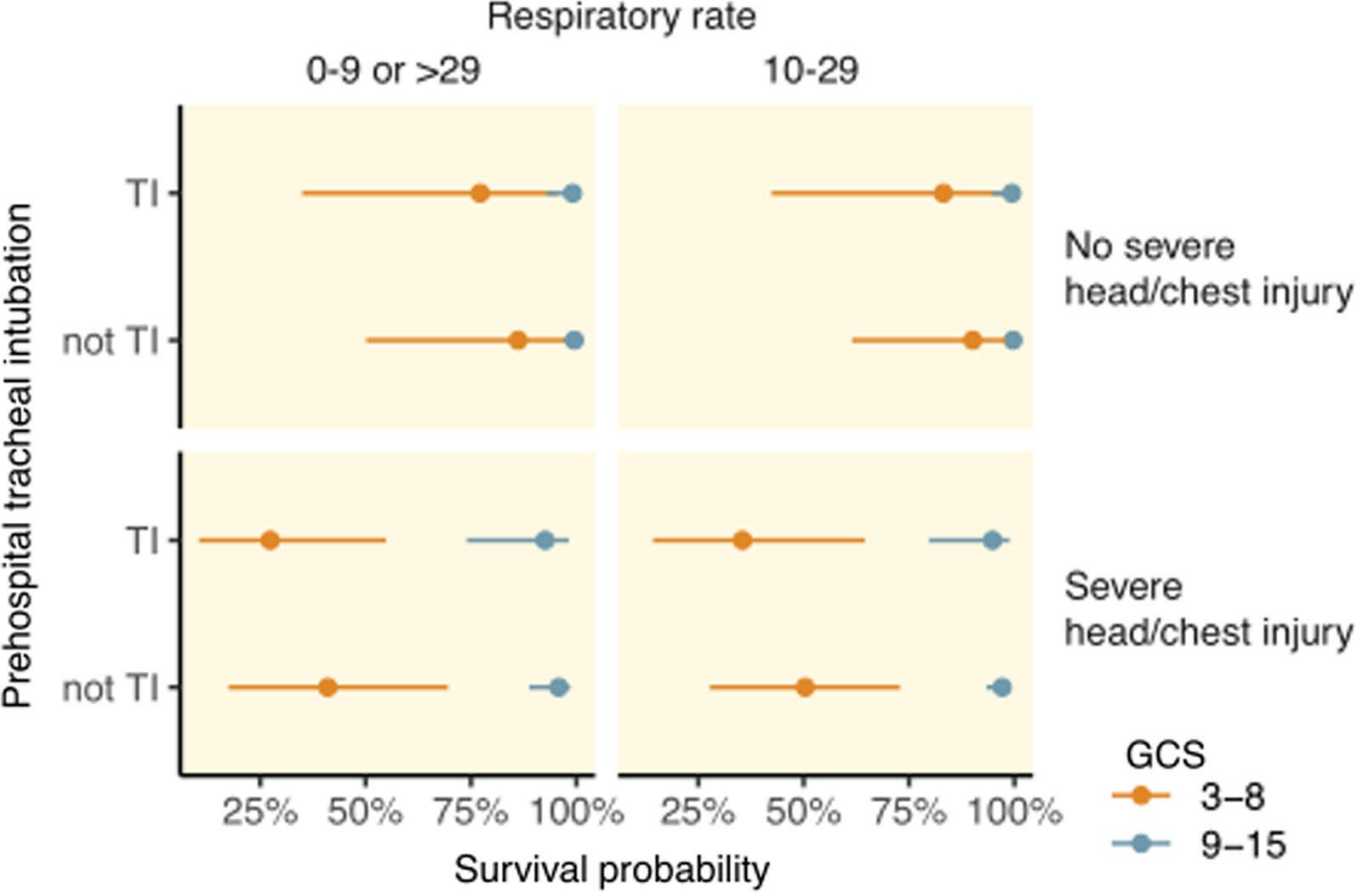
Regression model for mortality associated with prehospital TI. Probabilities calculated using the median age (31 years). Abbreviation: GCS = Glasgow coma scale

The 30-day mortality was 76% (*n* = 90) for patients with prehospital TI, 21% (*n* = 117) for patients without prehospital TI, 42% (*n* = 105) among patients with TI in the ED, and 22% (*n* = 107) for patients without TI in the ED (Table 3). Prehospital TI was associated with a higher 30-day mortality rate than no prehospital intubation, OR 12.4 (CI 7.82, 20.1, *p* < 0.001), which was reduced when adjusted for ISS, OR 7.84 (CI 4.68, 13.4, *p* < 0.001). Survival as a function of ISS in patients with and without prehospital TI is shown in Fig. 2. Adjusting for a GCS score ≤ 8 vs. > 8, an AIS head and/or thoracic injury score ≥ 3 vs. < 3, and a RR ≤ 9/ > 29 vs. 10–29 further reduced the associated mortality of prehospital TI when compared with no prehospital TI (OR 1.85, CI 0.49, 6.96), and the association was not significant (*p* = 0.37) (Fig. 3). Prehospital TI was associated with an increased mortality OR 4.26 (CI 2.57, 7.27, *p* < 0.001) compared with TI in the ED, even after adjustment for ISS OR 2.88 (CI 1.64, 5.14, *p* < 0.001). The Glasgow Outcome Scale (GOS) score was generally higher for patients who were intubated in the ED than for those with prehospital TI.

### Prehospital provider level

**Fig. 4:**
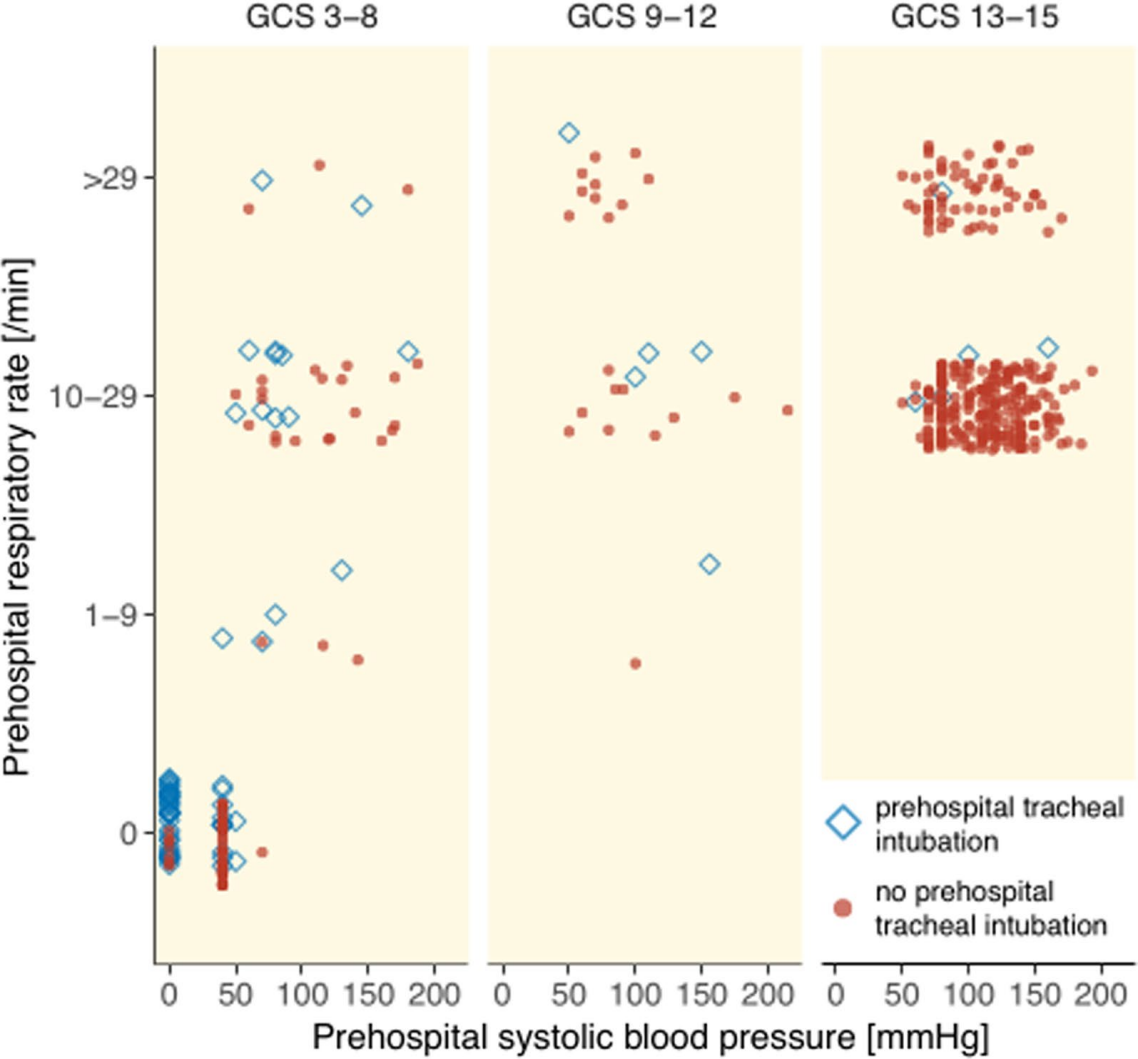
Patient characteristics associated with prehospital TI. Abbreviation: GCS = Glasgow coma scale

The majority, 54.2% (*n* = 64), of prehospital TI were performed by physicians with an associated mortality of 70% (*n* = 45), and 44.9% (*n* = 53) were intubated by nurses with a mortality of 85% (*n* = 45). The median ISS was 32 (IQR 24, 59) for patients intubated by physicians and 35 (IQR 26, 75) for patients intubated by nurses. The most common characteristics associated with prehospital TI were hemodynamic collapse (≤ 40 mmHg) and low GCS score (≤ 8) (Fig. 4).

### Traumatic cardiac arrests

The majority (99%) of patients with a SBP ≤ 40 mmHg and a GCS score ≤ 8 suffered TCA. In total, 18.9% (*n* = 154) of patients were registered as TCA, of whom 50% (*n* = 77) were prehospitally intubated and 36.4% (*n* = 56) were intubated in the ED. Nine patients (5.8%) who suffered TCA survived: 3 with prehospital TI, 5 with intubation in the ED, and 1 without TI.

### Traumatic cardiac arrests excluded

**Fig. 5:**
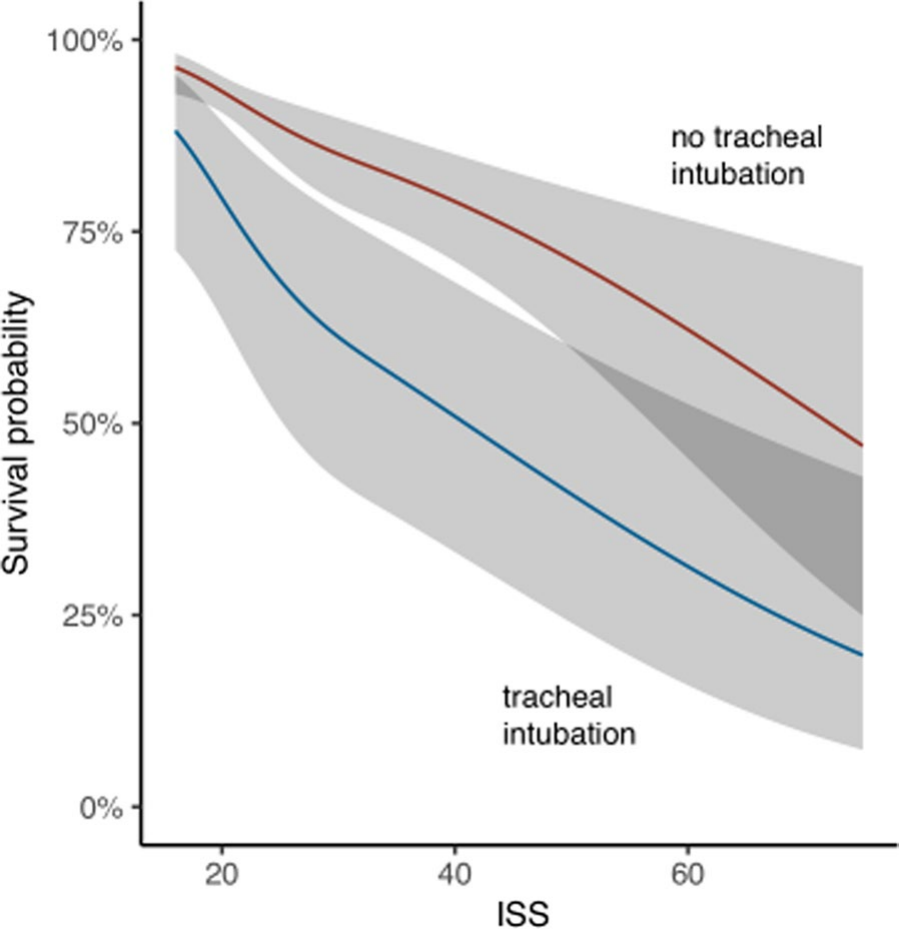
Mortality associated with prehospital TI and ISS with traumatic cardiac arrests excluded. Abbreviation: ISS = injury severity score

Patient characteristics and outcomes with TCA excluded are presented in Table 4. GSW (50%) was the most frequent injury mechanism for prehospital TI compared with SW (57%) for intubations in the ED. The median ISS was 26 (IQR 22, 39) for prehospital TI and 25 (IQR 18, 29) for TI in the ED. The majority (67%) of prehospital TI had a GCS score 3–8, compared with 25% of TI in the ED. The median systolic blood pressure was 100 mmHg (IQR 85, 145) for prehospital TI and 118 mmHg (IQR 95, 130) for intubations in the ED; 33% of prehospital TI had a systolic blood pressure of ≤ 90 mmHg compared with 23% of patients intubated in the ED. The mortality rate was 38% (*n* = 15) for prehospital TI, 9.2% (*n* = 45) for patients without prehospital TI, 23% (*n* = 35) for TI in the ED and 6.5% (*n* = 21) for patients without TI in the ED. There was no significantly increased mortality associated with prehospital TI with TCA excluded (OR 2.07 [CI 0.93, 4.51, *p* = 0.068]) compared with intubation in the ED, with OR 1.39 (CI 0.56, 3.26, *p* = 0.5) when adjusting for ISS. When TCAs were excluded, prehospital TI had a significantly increased mortality compared with patients without prehospital TI (OR 5.91, CI 2.86, 11.9 [*p* < 0.001]), even when adjusting for ISS (OR 3.28, CI 1.46, 7.11 [*p* = 0.003]) (Fig. 5). A flow chart for prehospital and ED TI with TCA excluded can be found in Additional file 1. A regression model for mortality associated with prehospital TI with TCA excluded can be found in Additional file 2. Patient characteristics associated with prehospital TI with TCA excluded can be found in Additional file 3.

**Table 4:** Traumatic cardiac arrests excluded

Characteristic	Prehospital airway management		ED airway management	
	TI, N = 40^*1*^	not TI, N = 488^*1*^	TI, N = 155^*1*^	not TI, N = 322^*1*^
Age (years)	37.5 (22.3, 55.3)	31 (23, 46)	31 (24, 46.8)	30 (22, 45.8)
(Missing)	0	1	1	0
Sex				
Female	4/40 (10%)	44/488 (9.0%)	12/155 (7.7%)	31/322 (9.6%)
Male	36/40 (90%)	444/488 (91%)	143/155 (92%)	291/322 (90%)
Injury mechanism				
GSW	20/40 (50%)	135/488 (28%)	55/155 (35%)	85/322 (26%)
SW	16/40 (40%)	312/488 (64%)	89/155 (57%)	204/322 (63%)
Other	4/40 (10%)	41/488 (8.4%)	11/155 (7.1%)	33/322 (10%)
ISS	26 (22, 39)	19 (17, 26)	25 (18, 29)	19 (17, 26)
Prehospital GCS score				
3–8	22/33 (67%)	39/396 (9.8%)	30/122 (25%)	23/269 (8.6%)
9–12	5/33 (15%)	25/396 (6.3%)	17/122 (14%)	10/269 (3.7%)
13–15	6/33 (18%)	332/396 (84%)	75/122 (61%)	236/269 (88%)
(Missing)	7	92	33	53
First blood pressure (mmHg)	100 (85, 145)	120 (100, 138)	118 (95, 130)	119.5 (100, 140)
(Missing)	19	163	76	94
First blood pressure (RTS)				
No carotid	0/15 (0%)	1/120 (0.8%)	0/52 (0%)	1/68 (1.5%)
Only carotid	2/15 (13%)	9/120 (7.5%)	8/52 (15%)	1/68 (1.5%)
Femoral	3/15 (20%)	12/120 (10%)	6/52 (12%)	7/68 (10%)
Weak radial	4/15 (27%)	42/120 (35%)	19/52 (37%)	24/68 (35%)
Clear radial	6/15 (40%)	56/120 (47%)	19/52 (37%)	35/68 (51%)
First blood pressure ≤ 90 mmHg	60/325 (18%)	7/21 (33%)	40/228 (18%)	18/79 (23%)
(Missing)	163	19	94	76
First respiratory rate (RTS)				
0	0/24 (0%)	1/432 (0.2%)	0/128 (0%)	1/280 (0.4%)
1–9	3/24 (12%)	4/432 (0.9%)	2/128 (1.6%)	4/280 (1.4%)
10–29	16/24 (67%)	323/432 (75%)	89/128 (70%)	212/280 (76%)
> 29	5/24 (21%)	104/432 (24%)	37/128 (29%)	63/280 (22%)
(Missing)	16	56	27	42
Outcomes				
Ventilator days	3 (1, 5.5)	1 (1, 3)	1 (1, 4)	1 (1, 3)
(Missing)	9	243	19	212
Glasgow Outcome Scale score				
1	15/38 (39%)	44/488 (9%)	36/155 (23%)	19/321 (5.9%)
2	0/38 (0%)	4/488 (0.8%)	2/155 (1.3%)	2/321 (0.6%)
3	11/38 (29%)	83/488 (17%)	36/155 (23%)	49/321 (15%)
4	9/38 (24%)	216/488 (44%)	56/155 (36%)	152/321 (47%)
5	2/38 (5.3%)	136/488 (28%)	23/155 (15%)	96/321 (30%)
Unknown	1/38 (2.6%)	5/488 (1%)	2/155 (1.3%)	3/321 (0.9%)
(Missing)	2	0	0	1
30-day survival				
Dead	15/40 (38%)	45/488 (9.2%)	35/155 (23%)	21/322 (6.5%)
Alive	23/40 (57%)	435/488 (89%)	116/155 (75%)	296/322 (92%)
Unknown	2/40 (5%)	8/488 (1.6%)	4/155 (2.6%)	5/322 (1.6%)

^*1*^*Median (IQR); n/N (%).* Abbreviations: ED = emergency department, GSW = gunshot wounds, IQR = interquartile range, SGA = supraglottic airway device, SW = stab wounds, TI = tracheal intubation

## Discussion

In this observational multicenter study, we showed that prehospital TI was associated with a higher 30-day mortality rate than intubations performed in the ED, even after adjustment for ISS. This was specifically related to TCA, and in patients without cardiac arrest, prehospital TI did not affect the mortality rate when compared with intubations in the ED. Previous studies have shown that increased mortality was associated with prehospital advanced airway management (69.2%) when compared with in-hospital airway management (35.9%) in patients with penetrating trauma.[16] Taghavi et al. found prehospital TI to be the strongest predictor of mortality in patients with penetrating trauma (OR 11.88, *p* < 0.001) compared with ISS (OR 1.07) and GSW (OR 7.08).[23] A meta-analysis of undifferenced trauma with TCA excluded observed an increased mortality of 48% with prehospital TI compared to 29% for TI in the ED, with an OR of 2.59 when adjusted for ISS.[9] We showed that this association was also present in the Swedish context. However, no difference in mortality was detected when excluding patients with TCA (OR 2.07, *p*  = 0.068). The rationale for this subgroup analysis was the finding that the majority (64%) of prehospital TI was performed without medication, compared with 16% in the ED, as TI without medication is likely to be performed in a situation of cardiac arrest.

A total of 154 (18.9%) patients suffered from TCA, 133 (86.4%) of whom required TI either prehospital or in the ED, and 9 (5.8%) patients survived. The frequency of cardiac arrests was substantially higher than previously reported.[14, 17] As registration in SweTrau is restricted to patients arriving at participating hospitals, the high frequency of TCA may represent an unwillingness to terminate prehospital resuscitation efforts in a cohort of young patients, but the cause is unclear and requires further study. Heffner et al. found cardiac arrest as a complication of anesthesia in 12% of cases in a mixed cohort of medical and trauma patients with hypotension, and Stausberg et al. observed TCA in 3.2% of all patients in a cohort of severely injured trauma patients managed by physicians.[14, 17] Excluding TCA reduced the difference in ISS and systolic blood pressure between prehospital and ED intubations, although the GCS score in patients with prehospital TI remained substantially lower than those with intubations in the ED. It is known that patients who require prehospital TI have higher ISS than those with TI in the ED [9, 16, 24, 25], which we could confirm in our cohort (ISS 33 versus 25). It is likely that a higher ISS predisposed patients to a higher risk of TCA. We could not determine whether the TCAs were preexistent or occurred after anesthesia. Systolic hypotension and shock prior to anesthesia induction have been observed as risk factors for cardiac arrest.[13, 14] In addition, anesthesia and positive pressure ventilation in patients with hemorrhagic shock are debated and may have contributed to the observed increase in mortality in patients with prehospital TI where the majority of intubated patients were in circulatory collapse or shock.[14, 26, 27] Furthermore, positive pressure ventilation decreases venous return and cardiac output.[28, 29] Hemorrhagic shock represents a low flow state with reduced capacity for transportation of produced CO_2_, and normal respiratory rates may reduce pCO_2_ with subsequent further reductions in venous return, mean arterial pressure and cardiac output.[13, 25].

Patient parameters associated with prehospital TI were analyzed using multivariate models, with and without TCA. Hemodynamic collapse (≤ 40 mmHg) and low GCS score (≤ 8) were the parameters most associated with prehospital TI with TCA included, compared with low GCS score (≤ 8) and hypotension (≤ 90 mmHg) with TCA excluded. We have previously shown that a substantial proportion of these patients suffered head injuries, which can also be reflected in the low GCS score.[18] Hypotension, related to injury or anesthesia, may exacerbate existing brain injury and contribute to increased mortality.[30] The correct indication and timing of TI in penetrating trauma are still under discussion.[9, 13, 16] Prehospital TI is an experience dependent, hazardous procedure that can and should be performed according to in-hospital standards.[11, 12, 31] Prehospital TI may increase the scene time which have been associated with an increased mortality[32], although experienced providers are capable of administering prehospital anesthesia in patients with hemorrhagic shock with short scene time.[13, 17, 33] The scene time was significantly longer (median 21 versus 12 min) when prehospital TI was required, which may have contributed to the increased mortality, although this is not clear. It is likely that the severity of the clinical situation required TI, and the observed increase in mortality reflected severely injured patients. High mortality in patients receiving prehospital TI was observed both when performed by physicians (70%) and nurses (85%). When TCA was excluded, the mortality was reduced for both physicians (30%) and nurses (50%). In comparison, Stausberg et al. observed increased mortality associated with prehospital TI in a physician-based emergency medical service (EMS), and Fevang et al. observed increased mortality associated with prehospital TI in trauma patients despite similar first pass success rates.[9, 17] While quality indicators of airway management (such as first pass success, etc.) are not registered in SweTrau, our results suggest that increased mortality associated with prehospital TI in trauma patients was not primarily driven by the experience of airway providers.

Prehospital anesthesia in hemodynamically unstable penetrating trauma patients is challenging. Emergency medical services are heterogeneous in organization and available competence, which therefore complicate comparisons.[34–37] Notably, Anglo-American EMS teams are largely comprised of paramedics while EMS teams in parts of Europe are a physician-based system.[34, 36, 38] Each ambulance in Sweden is staffed with a registered or specialist nurse, and several regions have access to physician-staffed, second tier EMS units.[39] Several interventions involving these second-tier EMS units have recently been initiated to counteract the challenges associated with severe, penetrating trauma. These include blood transfusions during anesthesia in trauma patients with hemorrhagic shock and a focus on shortening the scene time.[33] The effects of these new interventions in the Swedish context will be analyzed in future studies.

This study has some limitations to be discussed. First, it is an observational study with inherent biases, and inferences regarding the causation of prehospital TI and mortality could not be made. Second, SweTrau is primarily a trauma registry, and quality indicators of airway management were not registered, which limits the analysis. Third, the coverage of SweTrau increased during the study period, which could confound incidence. However, we did not analyze incidence trends. Fourth, the transcription of ordinal data (blood pressure) limits the multivariate analysis, which was primarily intended as a visualization of associations. Fifth, as registration in SweTrau was limited to patients who arrived at a participating hospital, prehospital deaths are a possible source of selection bias.

## Conclusion

Prehospital TI was associated with a higher mortality rate than intubations performed in the ED, which was related to TCA; intubation did not affect mortality in patients without cardiac arrest. The mortality rate was substantial when airway management was needed, regardless of TCA, demonstrating the lethality of severe, penetrating trauma and the challenges posed when anesthesia is needed. Several interventions, including whole blood transfusions, implementation of second-tier EMS units and measures to shorten the scene time, have been initiated to counteract challenges with penetrating trauma in Sweden. The effects of these new interventions will be analyzed in future studies.

### Supplementary Information

**Additional file 1: Figure 6.** Flowchart of TI with traumatic cardiac arrests excluded. Abbreviations: ED = emergency department, ISS = injury severity score, TCA = traumatic cardiac arrest, TI = tracheal intubation.

**Additional file 2: Figure 7.** Regression model for mortality associated with prehospital TI with traumatic cardiac arrests excluded. Probabilities calculated using the median age (31 years). Abbreviation: GCS = Glasgow coma scale.

**Additional file 3: Figure 8.** Patient characteristics associated with prehospital TI with traumatic cardiac arrests excluded. Abbreviation: GCS = Glasgow coma scale.

## Data Availability

The dataset analyzed during the current study is available in the SweTrau registry, [https://rcsyd.se/swetrau/].

## Abbreviations

AIS: Abbreviated injury scale
ED: Emergency department
EMS: Emergency medical service
GCS: Glasgow coma scale
GSW: Gunshot wound
ISS: Injury severity score
OR: Odds ratio
RR: Respiratory rate
SGA: Supraglottic airway device
SIR: Swedish intensive care registry
SW: Stab wound
SweTrau: Swedish trauma registry
TCA: Traumatic cardiac arrest
TI: Tracheal intubation

## References

[1] Collaborators GCoD. Global, regional, and national age-sex-specific mortality for 282 causes of death in 195 countries and territories, 1980-2017: a systematic analysis for the Global Burden of Disease Study 2017. Lancet. 2018;392(10159):1736-88. doi: 10.1016/S0140-6736(18)32203-7. 10.1016/S0140-6736(18)32203-7 PMC6227606 30496103

[2] Wihlke G, Strömmer L, Troëng T, Brattström O (2021). Long-term follow-up of patients treated for traumatic injury regarding physical and psychological function and health-related quality of life. Eur J Trauma Emerg Surg.

[3] Sheffy N, Chemsian RV, Grabinsky A (2014). Anaesthesia considerations in penetrating trauma. Br J Anaesth.

[4] Descamps C, Hamada S, Hanouz JL, Vardon-Bounes F, James A, Garrigue D (2022). Gunshot and stab wounds in France: descriptive study from a national trauma registry. Eur J Trauma Emerg Surg.

[5] The Swedish National Council for Crime Prevention. Murder and Manslaughter. (2021). Available online at: https://bra.se/bra-in-english/home/publications/archive/publications/2021-05-26-gun-homicide-in-sweden-and-other-european-countries.html.

[6] Teixeira PG, Inaba K, Hadjizacharia P, Brown C, Salim A, Rhee P (2007). Preventable or potentially preventable mortality at a mature trauma center. J Trauma.

[7] Esposito TJ, Sanddal ND, Hansen JD, Reynolds S (1995). Analysis of preventable trauma deaths and inappropriate trauma care in a rural state. J Trauma.

[8] Braithwaite S, Stephens C, Remick K, Barrett W, Guyette FX, Levy M (2022). Prehospital Trauma airway management: an NAEMSP position statement and resource document. Prehosp Emerg Care.

[9] Fevang E, Perkins Z, Lockey D, Jeppesen E, Lossius HM (2017). A systematic review and meta-analysis comparing mortality in pre-hospital tracheal intubation to emergency department intubation in trauma patients. Crit Care.

[10] Pepe PE, Roppolo LP, Fowler RL (2015). Prehospital endotracheal intubation: elemental or detrimental?. Crit Care.

[11] Gellerfors M, Fevang E, Bäckman A, Krüger A, Mikkelsen S, Nurmi J (2018). Pre-hospital advanced airway management by anaesthetist and nurse anaesthetist critical care teams: a prospective observational study of 2028 pre-hospital tracheal intubations. Br J Anaesth.

[12] Crewdson K, Rehn M, Brohi K, Lockey DJ (2018). Pre-hospital emergency anaesthesia in awake hypotensive trauma patients: beneficial or detrimental?. Acta Anaesthesiol Scand.

[13] Hudson AJ, Strandenes G, Bjerkvig CK, Svanevik M, Glassberg E (2018). Airway and ventilation management strategies for hemorrhagic shock. To tube, or not to tube, that is the question. J Trauma Acute Care Surg..

[14] Heffner AC, Swords DS, Neale MN, Jones AE (2013). Incidence and factors associated with cardiac arrest complicating emergency airway management. Resuscitation.

[15] Lockey DJ, Healey B, Crewdson K, Chalk G, Weaver AE, Davies GE (2015). Advanced airway management is necessary in prehospital trauma patients. Br J Anaesth.

[16] Rajani RR, Ball CG, Montgomery SP, Wyrzykowski AD, Feliciano DV (2009). Airway management for victims of penetrating trauma: analysis of 50,000 cases. Am J Surg.

[17] Stausberg T, Ahnert T, Thouet B, Lefering R, Böhmer A, Brockamp T (2022). Endotracheal intubation in trauma patients with isolated shock: universally recommended but rarely performed. Eur J Trauma Emerg Surg.

[18] Günther M, Dahlberg M, Rostami A, Azadali A, Arborelius UP, Linder F (2021). Incidence, demographics, and outcomes of penetrating trauma in sweden during the past decade. Front Neurol.

[19] Brohi K (2008). The Utstein template for uniform reporting of data following major trauma: a valuable tool for establishing a pan-European dataset. Scand J Trauma Resusc Emerg Med.

[20] Anual Report from the Swedish Trauma Registry (SweTrau) 2019. [Internet]. 2020.

[21] Rehn M, Hyldmo PK, Magnusson V, Kurola J, Kongstad P, Rognås L (2016). Scandinavian SSAI clinical practice guideline on pre-hospital airway management. Acta Anaesthesiol Scand.

[22] Deakin CD, Low JL (2000). Accuracy of the advanced trauma life support guidelines for predicting systolic blood pressure using carotid, femoral, and radial pulses: observational study. BMJ.

[23] Taghavi S, Vora HP, Jayarajan SN, Gaughan JP, Pathak AS, Santora TA (2014). Prehospital intubation does not decrease complications in the penetrating trauma patient. Am Surg.

[24] Shafi S, Gentilello L (2005). Pre-hospital endotracheal intubation and positive pressure ventilation is associated with hypotension and decreased survival in hypovolemic trauma patients: an analysis of the National Trauma Data Bank. J Trauma.

[25] Wang HE, Brown SP, MacDonald RD, Dowling SK, Lin S, Davis D (2014). Association of out-of-hospital advanced airway management with outcomes after traumatic brain injury and hemorrhagic shock in the ROC hypertonic saline trial. Emerg Med J.

[26] Heffner AC, Swords D, Kline JA, Jones AE (2012). The frequency and significance of postintubation hypotension during emergency airway management. J Crit Care..

[27] Kim WY, Kwak MK, Ko BS, Yoon JC, Sohn CH, Lim KS (2014). Factors associated with the occurrence of cardiac arrest after emergency tracheal intubation in the emergency department. PLoS One.

[28] Downs JB, Douglas ME, Sanfelippo PM, Stanford W, Hodges MR (1977). Ventilatory pattern, intrapleural pressure, and cardiac output. Anesth Analg.

[29] Pepe PE, Lurie KG, Wigginton JG, Raedler C, Idris AH (2004). Detrimental hemodynamic effects of assisted ventilation in hemorrhagic states. Crit Care Med.

[30] Rakhit S, Nordness MF, Lombardo SR, Cook M, Smith L, Patel MB (2021). Management and challenges of severe traumatic brain injury. Semin Respir Crit Care Med..

[31] Lossius HM, Røislien J, Lockey DJ (2012). Patient safety in pre-hospital emergency tracheal intubation: a comprehensive meta-analysis of the intubation success rates of EMS providers. Crit Care.

[32] Jakob DA, Müller M, Jud S, Albrecht R, Hautz W, Pietsch U (2023). The forgotten cohort-lessons learned from prehospital trauma death: a retrospective cohort study. Scand J Trauma Resusc Emerg Med.

[33] Árnason B, Hertzberg D, Kornhall D, Günther M, Gellerfors M (2021). Pre-hospital emergency anaesthesia in trauma patients treated by anaesthesiologist and nurse anaesthetist staffed critical care teams. Acta Anaesthesiol Scand.

[34] National Association of State EMS Officials. National EMS Scope of Practice Model 2019. National Highway Traffic Safety Administration, US Department of Transportation. 2019.

[35] Tjelmeland IBM, Masterson S, Herlitz J, Wnent J, Bossaert L, Rosell-Ortiz F (2020). Description of Emergency Medical Services, treatment of cardiac arrest patients and cardiac arrest registries in Europe. Scand J Trauma Resusc Emerg Med.

[36] Timmermann A, Russo SG, Hollmann MW (2008). Paramedic versus emergency physician emergency medical service: role of the anaesthesiologist and the European versus the Anglo-American concept. Curr Opin Anaesthesiol.

[37] Ong ME, Cho J, Ma MH, Tanaka H, Nishiuchi T, Al Sakaf O (2013). Comparison of emergency medical services systems in the pan-Asian resuscitation outcomes study countries: Report from a literature review and survey. Emerg Med Australas.

[38] Officials. NAoSE. National Association of State EMS Officials. National EMS Scope of Practice Model 2019. National Highway Traffic Safety Administration, US Department of Transportation. 2019.

[39] Khoshnood A (2020). The Swedish Ambulance Services. Eur J Emerg Med.

