# Supplementary material for: Prehospital and emergency department airway management of severe penetrating trauma in Sweden during the past decade
Source: Scand J Trauma Resusc Emerg Med. 2023 Nov 24;31:85. doi: 10.1186/s13049-023-01151-4 (PMC10675952; doi:10.1186/s13049-023-01151-4)
Supplement: Supplementary file 1 — Additional file 1: Figure 6. Flowchart of TI with traumatic cardiac arrests excluded. Abbreviations: ED = emergency department, ISS = injury severity score, TCA = traumatic cardiac arrest, TI = tracheal intubation. [file 13049_2023_1151_MOESM1_ESM.docx]

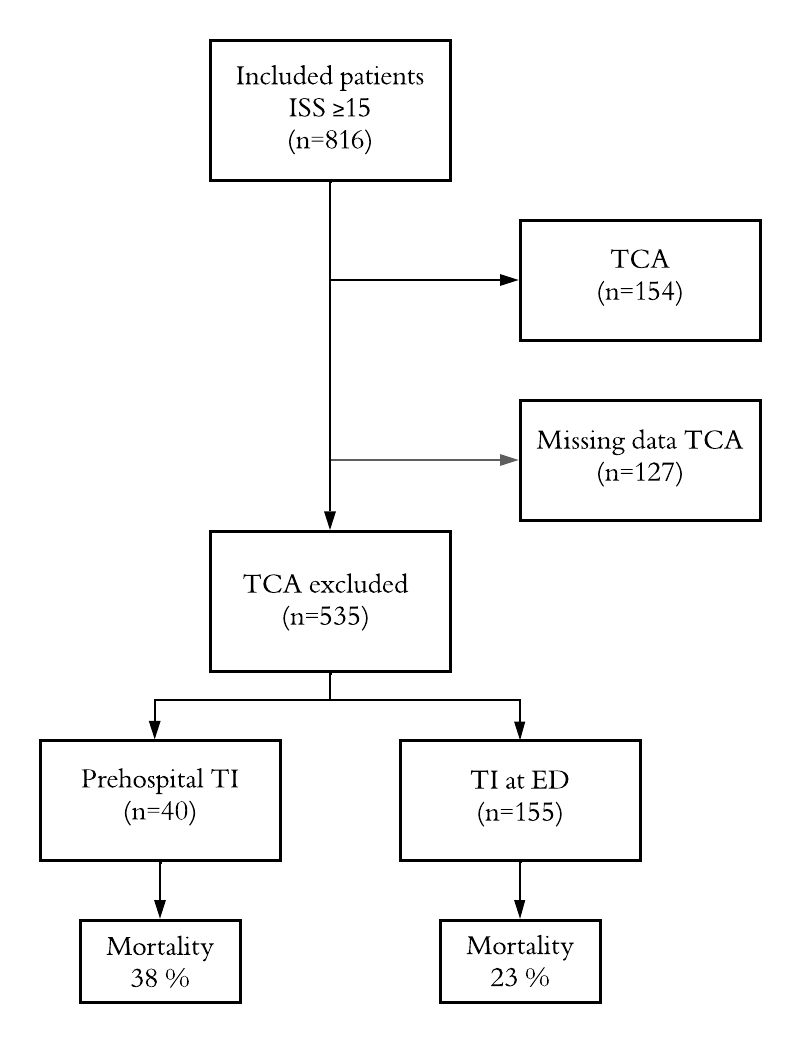


**Figure 6.** Flowchart of TI with traumatic cardiac arrests excluded. Abbreviations: ED = emergency department, ISS = injury severity score, TCA = traumatic cardiac arrest, TI = tracheal intubation.
