# Supplementary material for: Prehospital and emergency department airway management of severe penetrating trauma in Sweden during the past decade
Source: Scand J Trauma Resusc Emerg Med. 2023 Nov 24;31:85. doi: 10.1186/s13049-023-01151-4 (PMC10675952; doi:10.1186/s13049-023-01151-4)
Supplement: Supplementary file 2 — Additional file 2: Figure 7. Regression model for mortality associated with prehospital TI with traumatic cardiac arrests excluded. Probabilities calculated using the median age (31 years). Abbreviation: GCS = Glasgow coma scale. [file 13049_2023_1151_MOESM2_ESM.docx]

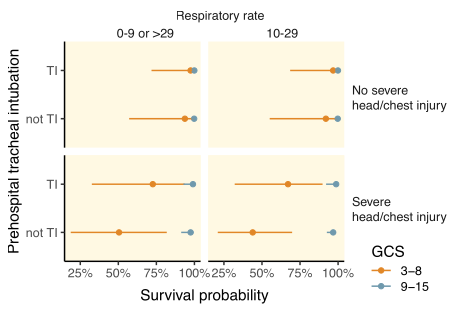


**Figure 7.** Regression model for mortality associated with prehospital TI with traumatic cardiac arrests excluded. Probabilities calculated using the median age (31 years). Abbreviation: GCS = Glasgow coma scale.
